# Metal Concentrations in Newcomer Women and Environmental Exposures: A Scoping Review

**DOI:** 10.3390/ijerph14030277

**Published:** 2017-03-08

**Authors:** Shirley X. Chen, Clare L. S. Wiseman, Dolon Chakravartty, Donald C. Cole

**Affiliations:** 1 Dalla Lana School of Public Health, University of Toronto, 155 College Street, Toronto, ON M5T 3M7, Canada; shirleyx.chen@mail.utoronto.ca (S.X.C.); dolon.chakravartty@mail.utoronto.ca (D.C.); donald.cole@utoronto.ca (D.C.C.); 2 School of the Environment, University of Toronto, 33 Willcocks Street, Toronto, ON M5S 3E8, Canada

**Keywords:** immigrant health, metals, environmental exposure, women’s health, review

## Abstract

Newcomer women from developing countries are recognized to be at risk for elevated exposures to environmental contaminants and associated negative health effects. As such, data on exposure sources and contaminant body burden concentrations is critical in the development of effective public health policies and interventions in support of newcomer health. We conducted a scoping review to gather evidence on important toxic metals of health concern, lead (Pb), mercury (Hg) and cadmium (Cd), and their concentrations and potential exposure sources among newcomer women. An initial 420 articles were identified through the databases MEDLINE, EMBASE and Scopus, many reporting by ethnicity rather than newcomer/immigrant status. Several articles reported metal concentrations for other biomarkers but did not include blood, nor stratify results. From the remainder, we selected a total of 10 articles for full textual review, which reported blood Pb, Hg or Cd levels for newcomer women and/or stratified blood metal results according to foreign birth or country of origin. Three of the articles reported higher Pb, Hg and Cd concentrations in newcomer women compared to their native-borne counterparts. Exposures identified as contributing to elevated Pb, Hg and Cd blood concentrations included: pica behaviour, the use of lead-glazed cookware or eye cosmetics, and fish/shellfish consumption. The review revealed a limited availability of data on metal body burden concentrations, exposure sources and routes among newcomer women specifically. More research is needed to better understand the extent to which newcomer women are disproportionately at risk of elevated metal exposures due to either country of origin or current exposures and to inform relevant, multi-national risk management strategies.

## 1. Introduction

Lead (Pb), mercury (Hg) and cadmium (Cd) are known toxic metals that pose a particular hazard to women and their children. For example, Cd may be more toxic to women compared to men [1]. Cadmium, Pb and Hg are all reproductive and developmental toxicants, at low, environmentally-relevant exposure levels [2]. Maternal blood Pb concentrations, for instance, are associated with negative birth outcomes such as intellectual impairment, low birth weight, pre-term delivery and spontaneous abortion [3,4,5,6,7]. Mercury is also a potent neurotoxicant, with demonstrated impaired effects on the cognitive and motoric functioning of children exposed in utero [1]. Similarly, Cd has also been identified as a potential neurodevelopmental toxicant at low exposure concentrations [8]. Cadmium exposures have also been associated with kidney damage and an elevated risk of osteoporosis and bone fractures in women [1].

Various factors have been identified which may contribute to metal exposures at concentrations of human health concern in women. Metal exposures and biomarker concentrations in women have been shown to be modulated by both gender and sex-related factors [9]. Biological mechanisms support an enhanced metal uptake following exposure and mobilization of bodily stores of toxicants, related to a woman’s lifetime reproductive cycle including pregnancy, lactation and menopause [10,11]. For instance, women of reproductive age are at risk for iron-deficient anemia, which has been shown to be a strong predictor of elevated blood Pb and Cd concentrations [12,13]. It is important to note that the highest global rates of iron-deficient anemia among women of reproductive age has been reported for the South-East Asia Region (42%), followed by the African Region (39%) [14]. By comparison, women of reproductive age in North America have a reported prevalence of 17%, which emphasizes the importance of considering country or region of origin as a potential risk factor for Cd and Pb concentrations among newcomers.

Gender-related practices, including the use of imported cosmetics, medicines/herbal treatments and personal care products, may also be important sources of toxic metal exposures among newcomer women. Women using traditional Asian herbal supplements in the United States, for instance, have been reported to have significantly elevated blood Pb concentrations relative to non-users [15]. Imported personal care products and cosmetics such as skin-lightening creams and kohl have also been identified as important sources of Hg and Pb [16,17]. Other sources of Pb exposures for populations residing in middle to low income countries include emissions associated with artisanal mining and processing activities, toxic waste sites, leaded paint and remaining leaded gas use [18,19,20,21]. Fish, shellfish and grains such as rice can be significant sources of Hg exposures [22,23]. Methyl mercury (MeHg) contributes the bulk of Hg exposures in most individuals; notably through the consumption of long-living, predatory fish species [24]. Non-occupational Cd exposures occur mainly through Cd-containing food products and cigarette smoke [25,26].

There is a body of literature which shows that minorities may be at higher risk of elevated metal exposures. International studies have reported the occurrence of elevated exposures to metals among women of racial/ethnic minorities in developed countries [3,9,27,28]. In particular, the United States National Health and Nutrition Examination Survey (NHANES) stratifies by ethnicity in several categories: Non-Hispanic White, Non-Hispanic Black, Mexican American, Hispanic and Other. The “Other” group includes participants identifying as Asian, Pacific Islander, multi-racial or a race/ethnicity other than the above mentioned groups. Studies have found Pb biomarker concentrations to be the highest among Non-Hispanic Black women and Mexican American women [15,29,30]. Mercury and Cd have been reported to be the highest in the “Other” group [31,32,33,34,35,36]. However, data on ethnic/racial minorities in the USA includes large numbers of women born in the USA, making extrapolation to newcomers difficult.

Additional factors which may contribute to elevated toxic metal body burden concentrations among newcomers include coming from a country where environmental regulations may be less stringent and public health policies that are protective of human health less common [27]. The continued consumption of traditional diets and use of imported products, combined with a greater likelihood of living in impoverished conditions in their new home countries, may also contribute to elevated exposures post-migration. The existence of elevated metal concentrations among newcomer women relative to their native-borne counterparts post-migration reported elsewhere [37,38] may reflect disproportionate environmental exposures both before and following migration. Given the potential for negative health outcomes in both women and children, there is a need to identify potential toxic metal exposure sources that may place newcomer women at increased risk to enable host countries to develop effective interventions.

The purpose of this scoping review was to systemically assess the current state of knowledge on metal concentrations and possible environmental exposure sources in newcomer women. The specific objectives were: (1) to examine the available data on blood Pb, Hg and Cd levels in women who have migrated to high income countries; and (2) to gather information on identified environmental sources of exposures to these metals, including those that occur pre- and post-migration to host countries, as reported in peer-reviewed literature.

## 2. Methods

A scoping review method [39] was used to identify relevant literature. We identified articles through the databases MEDLINE, EMBASE and Scopus using the following search terms: newcomer*/immigra*/refugee*/migra* AND lead/Pb/mercury/Hg/Cd/cadmium/metal*/“Heavy Metal” AND blood/urin*/hair*/nail*/biomark*/“biological marker”. The following limits were placed on the search: “English”, “female”, “adult”, “humans” and “2006 to present”. Several iterations of the search were conducted between January and October 2016 to ensure that all relevant literature was captured for the review.

We scanned article abstracts, keywords and titles to assess their relevance for full-text review. Articles were only considered if they: (1) were published in peer-reviewed journals; (2) presented primary research conducted on women (defined as >18 years of age); and (3) reported body burdens for Pb, Hg and/or Cd for newcomer populations or stratified metal concentrations according to foreign birth. We define newcomer women in this review broadly as women who are foreign-born, have migrated to a country where they were not born whether temporarily or permanently, regardless of citizenship or duration of residence since migration [40]. This includes those falling under the commonly defined categories of immigrant, refugee and migrant. Literature reviews, case studies and abstracts were not considered and articles were excluded if they focused exclusively on: (1) exposures and concentrations in men and/or children; (2) occupational exposures; and (3) women of ethnic minorities in high income countries without specification of foreign birth.

The articles that we selected for full-text review were examined and blood Pb, Hg and Cd concentrations in immigrant women extracted. We also extracted details regarding the specific characteristics of the study populations examined in the respective studies, including those relating to study location, sample size, ethnicity/race, age and country of origin. Identified environmental exposures and sources, such as those related to diet and cultural practices, were noted. Research gaps and priorities were synthesized from discussion sections of included articles and our own reflections on needs in the field.

## 3. Scoping Review Results

**Figure 1:**
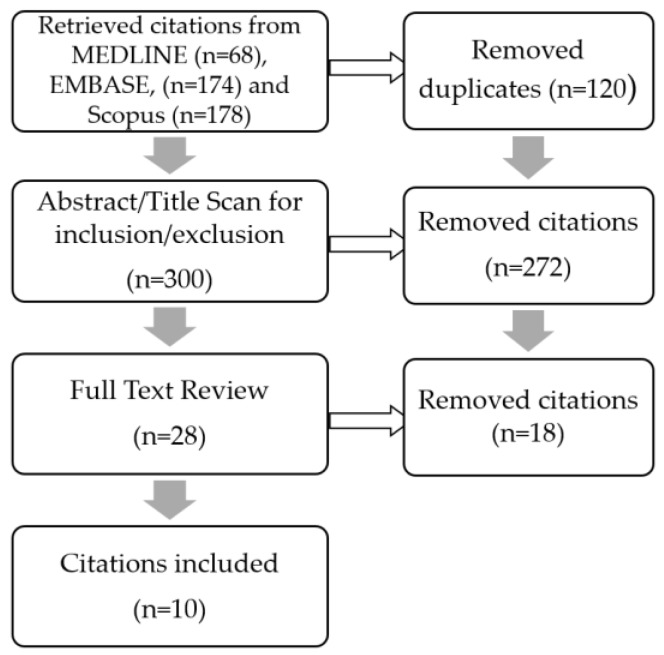
Scoping review article selection process.

The selection process of the scoping review is outlined in Figure 1. We initially retrieved a total of 420 articles retrieved from MEDLINE (*n* = 68), EMBASE (*n* = 174) and Scopus (*n* = 178). Once duplicates were removed, the titles, abstracts and keywords of the remaining 300 articles were scanned by three reviewers for their relevance. Agreement among the reviewers of the initial scan was high (kappa = 0.93) across reviewer pairs on article selection for the review. We settled inconsistencies with a second round of scanning and consensus was achieved regarding relevance for the full-text review for all articles between all authors.

We selected a total of 28 articles for further examination after a review of the titles and abstracts. Of these, 10 articles which met our inclusion/exclusion criteria were chosen to proceed to data extraction. Lead was the main metal examined, with eight studies investigating Pb body burdens. Three studies examined Hg concentrations, while only one surveyed blood Cd levels. All studies meeting the criteria for this review investigated blood as a biomarker for body burdens. Of the 18 articles that did not match the inclusion/exclusion criteria, most were excluded because the articles were reviews or the study population was stratified by race/ethnicity with no clear indication of newcomer status, as defined above.

The majority of studies were from the United States (*n* = 5), followed by Taiwan (*n* = 2) and Australia, Belgium and Canada at one apiece. Of the foreign-born women comprising the study populations, a total of 24 countries/regions of origin or ethnicities/races were represented, including Latina/Hispanic (*n* = 3 studies), China (*n* = 3), Mexico (*n* = 2), Other (*n* = 2), former USSR (*n* = 1), Poland (*n* = 1), Bulgaria (*n* = 1), Romania (*n* = 1), Albania (*n* = 1), Pakistan (*n* = 1), India (*n* = 1), Bangladesh (*n* = 1), Vietnam (*n* = 1), Mediterranean (*n* = 1), sub-Saharan African (*n* = 1), African American (*n* = 1), Caribbean or West Indian (*n* = 1), African (*n* = 1), Jamaica (*n* = 1), Dominican Republic (*n* = 1), Caucasian (*n* = 1), Japanese (*n* = 1), Filipino (*n* = 1), and Pacific Islander (*n* = 1).

### 3.1. Studies Investigating Pb Concentrations (n = 8)

Eight studies examined Pb concentrations in immigrant women (Table 1) [41,42,43,44,45,46,47]. Mean Pb levels, where reported, ranged from geometric means (GM) of 0.78 μg/dL (95% CI: 0.57, 1.10) in immigrants to Canada [37] to 29.5 μg/dL (95% CI: 25.9, 33.1) in immigrant Pb-poisoned women exhibiting pica behaviour in New York City, USA [41]. One study [47] did not report mean Pb levels but rather the proportion of foreign-born women with levels ≥20 μg/dL. Other than the two studies specifically examining women with elevated blood Pb levels (BLLs), no other studies reported BLLs exceeding the US Centre for Disease Control and Prevention (CDC) threshold. The American College of Obstetricians and Gynecologists outlines the U.S. CDC guidelines for screening and management for pregnant women with Pb exposures, with levels ≥5 μg/dL warranting follow-up to identify Pb exposure sources and potential abatement practices [48].

Among pregnant Latina women residing in an area of Albuquerque (NM, USA) and considered to be socially disadvantaged (87% of which were defined as “foreign-born”), few (<3%) were determined to have at risk BLLs of ≥3 μg/dL [42]. Only three of 140 participants had BLLs ≥3 μg/dL at 3.4 μg/dL, 5.6 μg/dL and 8.5 μg/dL. The (unspecified) mean BLL of the 39 study participants with samples analyzed using inductively coupled mass spectrometry (ICP-MS) was 1.06 (95% CI: 0.57, 1.55), with a range of 0.26–8.5 μg/dL [42]. Gulson et al. [43] reported BLLs of three groups of migrant women mostly of Eastern European origin in Australia. The geometric mean BLL for the study population combined was 2.73 μg/dL, with no significant difference between pregnant women (2.76 μg/dL) and non-pregnant migrants (2.77 μg/dL), well under the 5 μg/dL threshold.

Two studies investigated pregnant women with Pb poisoning [41,44]. Among pregnant women determined to be Pb-poisoned in New York, those reporting pica (*n* = 43/491) had the highest Pb levels at 29.5 μg/dL (95% CI: 25.9, 33.1) compared with non-pica eaters at 23.8 μg/dL (95% CI: 22.9, 24.7) [41]. Most of these women were classified as immigrants (98%), with the highest concentrations among those who originated from Mexico, followed by Jamaica and the Dominican Republic. A chart review of pregnant women presenting to a hospital in Brooklyn (New York, NY, USA) with BLL ≥ 10 μg/dL between 1999–2005 found that 100% of the women had a birthplace outside of the U.S. (44.9% born in Mexico, 26.9% in Pakistan, 12.3% other, 8.9% in Bangladesh, 6.7% in India) [44]. Forty-six percent of the women immigrated to the U.S. during their pregnancy. Mean BLLs were 16.82 μg/dL (95% CI: 14.82, 18.82) pre-intervention and 11.48 μg/dL post-nutritional supplementation and educational interventions.

The remaining studies, where reported, did not find average BLLs above the 5 μg/dL threshold. Three studies stratified their study population by foreign birth, comparing BLLs between immigrant and native women in Taiwan [45], stratifying cord blood Pb concentrations by mother’s country of origin in Belgium [47], and by foreign birth in Canada [37]. Wu et al. [45] reported higher BLLs in immigrant women from Vietnam, Mainland China, and Southeast Asia (2.23 μg/dL, 95% CI: 1.84, 2.62) compared to native women living in Taichung City, Taiwan (1.63 μg/dL, 95% CI: 1.41, 1.85, *p* = 0.04). Zhang et al. [47] found a higher proportion of maternal cord blood Pb levels exceeding 20 μg/dL in women originating from the Mediterranean (OR_adj_ = 5.51, 95% CI: 1.68, 18.08, *p* = 0.005) and sub-Saharan Africa (OR_adj_ = 8.14, 95% CI: 2.26, 29.40, *p* = 0.001). Lead values from Cycle 1 (2007–2009) of the Canadian Health Measures Survey (CHMS), Canada’s national biomonitoring survey, were reported for 16 foreign-born Canadians by Curren et al. [37] (BLL = 0.78 μg/dL, 95% CI: 0.57, 1.10). In comparison, 77 Canadian-born women were reported to have a mean Pb concentration of 0.57 μg/dL (95% CI: 0.53, 0.61).

Only one study [46] examined metal levels by stratifying based on years since arrival in country. Immigrant women to Taiwan (*n* = 239), 67 coming from mainland China, with the rest originating from elsewhere in southeast Asia, were grouped into two categories: (1) recent immigrants (≤5 years since arrival); and (2) less recent immigrants (>5 years since arrival). Blood Pb concentrations were reported among recent immigrants to Taiwan (2.67 μg/dL, 95% CI: 2.45, 2.89) and found to decrease with time since arrival in the country. Less recent immigrants had a mean BLL of 2.40 μg/dL (95% CI: 2.21, 2.59), while non-immigrants had the lowest BLL at 2.33 μg/dL (95% CI: 2.17, 2.49). As blood Pb levels of recent immigrants were significantly different from the non-immigrant population in Taiwan (*p* = 0.003), the authors suggested that blood Pb levels may decrease with time following immigration.

### 3.2. Studies Investigating Hg and Cd Concentrations (n = 3)

Three studies examined Hg concentrations in women (See Table 1) [37,49,50]. Mercury body burdens were reported as blood Hg [37], cord blood Hg [49,50] and urinary Hg concentrations [49]. No studies reported Hg levels exceeding 5.8 μg/L, the U.S. CDC’s guideline intervention level for women of childbearing age [51].

Curren et al. [37] compared blood Hg concentrations in Canadian foreign-born women (0.88 μg/L, 95% CI: 0.55, 1.40) to Canadian-born women (0.40 μg/L, 95% CI: 0.32, 0.50). In the same study, authors found higher Cd concentrations in Canadian foreign-born women (0.59 μg/L, 95% CI: 0.42, 0.83) compared to Canadian-born (0.46 μg/L, 95% CI: 0.38, 0.55). Geer et al. [49] described pregnant women in a predominantly immigrant community in the United States hailing mainly from the Caribbean or the West Indies. Mercury concentrations were 2.14 μg/L (GM, 95% CI: 1.76, 2.60) in cord blood and 0.45 μg/L (GM, 95% CI: 0.37, 0.55) in maternal urinary Hg. Soon et al. [50] studied pregnant women in a multiethnic maternal and child health cohort recruited at a major hospital in Hawaii. Approximately half (44%) of the women in this high fish consumer population had cord blood Hg levels ≥5 μg/L, with a mean blood Hg level of 5.20 µg/L (95% CI: 4.08, 6.33). 56.5% of Japanese women had cord blood levels of 5 μg/L and over, followed by Caucasian (42.9%), Chinese (34.8%), Hispanic (33.3%), then Filipina (28.1%) women.

### 3.3. Environmental Exposures Identified

Potential exposures to metals were considered in most of the articles in this review. Exposures to Pb included the use of kohl, particularly among sub-Saharan African women, and the use of traditional Tagine cooking vessels, a ceramic pottery used by women originating from the Mediterranean and North Africa [44]. Other identified environmental exposure sources include the use of Pb-glazed pottery cookware and residence in older housing [44]. In their study of pregnant Latina women, Bakhireva et al. [42] also found that pica behaviour was significantly associated with higher Pb levels. In Thihalolipavan’s et al. [41] study of Pb-poisoned pregnant women in New York City (*n* = 491), commonly consumed items included ceramics or brick (21%) and paint flakes or plaster materials (8%). About 30% of those with pica behaviour (*n* = 43) reported eating these items at least once per day. The observation that Mexican-born and Latina women were found to be more likely to exhibit pica tendencies highlights a need to consider for the development of risk communication messages and strategies that are culturally-sensitive and -relevant.

Wu et al. [45] observed that immigrants to Taiwan were more likely to work in Pb battery factories or other related industries such as metal smelting, solder, printing, or PVC and glass manufacturing, and have elevated Pb levels.

Not surprisingly, fish and seafood consumption was the most commonly identified source of elevated Hg exposures [49,50]. Japanese populations [50] were particularly identified as being at risk via this route of exposure. Around 91% of the surveyed Japanese participants ate fish and 42.9% had MeHg concentrations in cord blood ≥5 μg/L, representing the highest of both measures amongst all other countries of origin in the study. Interestingly, Japanese participants (*n* = 23) also consumed the least amount of fish in the last month of pregnancy at 9.6 ounces, compared to the next highest amount at 13.7 ounces in Filipino women (*n* = 32). More study participants had consumed yellowfin or bigeye tuna (ca. 50%) than any other fish type. The results indicate that Japanese women consume greater quantities of fish types known to be major sources of (MeHg) relative to those from other countries.

## 4. Discussion

Our review identified several biomonitoring studies (*n* = 10 studies), with limited comparability in methods of population selection and categorization. Most studies examined Pb and Hg; only one investigated Cd concentrations. Most reported mean Pb and Hg concentrations in newcomer women fell below the US CDC blood intervention level of 5 μg/dL and 5.8 μg/L for Pb and Hg in women of childbearing age, respectively [51]. However, in some of the examined studies, Pb [41,44] and Hg [50] concentrations bordered or exceeded levels of human health concern. This is of particular concern for women of reproductive age, given the potential for neurodevelopmental effects at such exposure concentrations in utero. Elevated blood metal concentrations were associated with such factors as recent immigration, low socioeconomic status and educational attainment [44], culturally-related practices [44] and dietary preferences reflective of country of origin [50].

Elevated metal concentrations were detected in foreign-born women among studies comparing metal levels between newcomer and non-newcomer women [37,45,46]. Future biomonitoring studies should report metal concentrations in newcomers, along with metal levels in native-born women to enable comparisons between these two groups. Additionally, it would be informative if studies would more clearly delineate the countries of origin for newcomer women to allow for the design of appropriate population-specific inventions.

Only one study examined time of residency as a factor for elevated metal levels in immigrant women [46]. Recency of immigration may be an important factor in body burdens of heavy metals as women are removed from potential exposures in their country of origin (e.g., leaded gasoline), or discontinue cultural practices putting them at higher risk of environmental exposures (e.g., use of lead-lined cookware). Conversely, it may be that these exposures are continuing post-migration. Time of residency in new home country or since arrival is an infrequently measured yet critically important factor to consider, especially in determining when and where exposures may have taken place.

Comparability between studies and analysis and interpretation of results was challenging due to reporting of blood metal levels, e.g., mean type not specified. Consistent distribution reporting (e.g., with percentiles) would help such syntheses. Many primary research studies had sample populations which were relatively small, reducing statistical power to detect differences between newcomer and non-newcomer populations. The substantial heterogeneity between sample newcomer populations prohibited the combined analysis of populations across studies. This review compliments existing studies of women of racial/ethnic minority women [15,29,30,31,32,33,34,35,36,52] recognizing the difficulty in delineating immigrants or newcomers among racial/ethnic stratifications. Our scoping review was limited precisely because of the limited availability of data distinguishing newcomers, highlighting the need for further research in a world where migration grows in importance.

## 5. Conclusions

This scoping review has identified a number of significant gaps in current knowledge about metal exposures among newcomer women. The identification of newcomer populations as a group with relatively elevated metal concentrations compared to native populations highlights a need for focused biomonitoring initiatives towards these understudied groups. Given the importance of toxic exposures and migration globally, studies in other net immigration countries would be of particular interest. Greater consideration of temporal factors is needed, including time since immigration as a variable of stratification. Finally, further research is required to document and understand sources of environmental exposure in newcomer women coming from varied cultural/ethnic/racial backgrounds. This improved knowledge would help guide and develop relevant, targeted interventions and inform risk management strategies both within a country and globally to protect the health of newcomer women.

## Figures and Tables

**Table 1:** Studies reporting biomarker lead, mercury and cadmium concentrations in newcomer women.

Reference	Metal of Interest	Biomarker Matrix	Study Design	Sample Size	Population	Country of Study	Race/Ethnicity	Mean Metal Level (95% CI)	Comparison Population (If Applicable)	Mean Comparison Metal Level (If Applicable)
Gulson et al. 2006 [43]	Pb	Blood	Cross-sectional	*n* = 39	Women of child bearing age, including pregnant women	Australia	Former Yugoslavia, former Soviet Union, Poland, Bulgaria, Romania, Albania, China	2.73 μg/dL		
Rastogi et al. 2007 [44]	Pb	Blood	Chart review	*n* = 89	Pregnant women with identified BLL ≥10 μg/dL	USA	Birthplace outside of USA: Mexico, Pakistan, India, Bangladesh, Others	Pre-interevention 16.82 μg/dL (14.82, 18.82) Post-intervention: 11.48 μg/dL		
Bakhireva et al. 2009 [42]	Pb	Blood	Cross-sectional	*n* = 140	Pregnant women <20 weeks of gestation	USA	Majority born outside of the U.S. Mainly Latina.	1.06 μg/dL (0.57, 1.55) (*n* = 39)		
Wu et al. 2009 [45]	Pb	Blood	Cross-sectional	*n* = 154	Immigrant women	Taiwan	Immigrant (Vietnam, Mainland China, Southeast China)	2.23 μg/dL (1.84, 2.62) (*n* = 71)	Native-born (non-immigrant)	1.63 μg/dL (1.41, 1.85) (*n* = 38)
Zhang et al. 2012 [47]	Pb	Umbilical cord blood	Cross-sectional	*n* = 220	Mothers who recently delivered in a maternity unit	Belgium	South Mediterranean, Sub-Saharan Africa	South-Mediterranean: 50% ≥20 μg/dL ^a^; Sub-Saharan Africa: 20% ≥20 μg/dL **^a^**	Western-European Women, Eastern-European Women	Western-European Women 30% ≥20 μg/L **^b^**, Eastern-European Women 0% ≥20 μg/L **^b^**
Geer et al. 2012 [49]	Hg	Umbilical cord blood/Urine	Cross-sectional	*n* = 190	Pregnant women in a predominantly immigrant community	USA	African American, Caribbean or West Indian, African, Latino/Hispanic	Cord blood **^b^**: 2.14 μg/L (1.76, 2.60) (*n* = 78) Maternal urinary mercury **^b^**^,**c**^: 0.45 μg/L (0.37, 0.55) (*n* = 183)		
Thihalolipavan et al. 2013 [41]	Pb	Blood	Chart review	*n* = 491	Lead poisoned pregnant women	USA	Mexican, Jamaican, Dominican Republic, Other	Pica eaters: 29.5 μg/dL (25.9, 33.1) Non-pica eaters: 23.8 μg/dL (22.9, 24.7)		
Wu et al. 2013 [46]	Pb	Blood	Cross-sectional	*n* = 239	Immigrant women	Taiwan	Immigrant	RI **^d^**: 2.67 μg/dL (2.45, 2.89) LRI **^e^**: 2.40 μg/dL (2.21, 2.59)	Native-born (non-immigrant)	NI **^f^**: 2.33 μg/dL (2.17, 2.49)
Curren et al. 2014 [37]	Pb, total Hg, Cd	Blood	Cross-sectional	*n* = 93	Canadian foreign-born women	Canada	Canadian foreign-born	Pb: 0.78 μg/dL **^b^** (0.57, 1.10) (*n* = 16) Hg: 0.88 μg/L **^b^** (0.55, 1.40) (*n* = 16) Cd: 0.59 μg/L **^b^** (0.42, 0.83) (*n* = 16)	Canadian native-born women	Pb: 0.57 μg/L **^b^** (0.53, 0.61) Hg: 0.40 μg/L **^b^** (0.32, 0.50) Cd: 0.46 μg/L **^b^** (0.38, 0.55)
Soon et al. 2014 [50]	MeHg	Umbilical Cord Blood	Secondary analysis of prospective cohort pilot study	*n* = 100	Multiethnic maternal and child cohort	USA	Hispanic, Caucasian, Japanese, Chinese, Filipino, Pacific Islander	5.20 μg/L (4.08, 6.33) % of women with cord blood ≥5 μg/L: Japanese—56.5% Caucasian—42.9% Chinese—34.8% Hispanic—33.3% Filipino—28.1%		

**^a^** Reported as % exceeding threshold concentration; **^b^** Metal concentrations reported as geometric means (non-specified in other cases); **^c^** creatinine corrected (μg/g); **^d^** RI = recent immigrant; **^e^** LRI = less recent immigrant; **^f^** NI = non-immigrant.

## References

[1] Vahter M., Åkesson A., Lidén C., Ceccatelli S., Berglund M. (2007). Gender differences in the disposition and toxicity of metals. Environ. Res..

[2] Rana S.V.S. (2014). Perspectives in endocrine toxicity of heavy metals—A review. Biol. Trace Elem. Res..

[3] Klitzman S., Sharma A., Nicaj L., Vitkevich R. (2002). Lead poisoning among pregnant women in New York City: Risk factors and screening practices. J. Urban Health.

[4] Borja-Arbuto V.H., Hertz-Picciotto I., Lopez M.R., Farias P., Rios C., Blanco J. (1999). Blood lead levels measured prospectively and risk of spontaneous abortion. Am. J. Epidemiol..

[5] Canfield R.L., Henderson C.R., Cory-Slechta D.A., Cox C., Jusko T.A., Lanphear B.P. (2003). Intellectual impairment in children with blood lead concentrations below 10 μg per deciliter. N. Engl. J. Med..

[6] Ernhart C.B. (2006). Effects of lead on IQ in children. Correspondence. Environ. Health Perspect..

[7] Lanphear B.P., Hornung R., Khoury J., Yolton K., Baghurst P., Bellinger D.C., Canfield R.L., Dietrich K.N., Bornschein R., Greene T. (2005). Low-level environmental lead exposure and children’s intellectual function: An international pooled analysis. Environ. Health Perspect..

[8] Ciesielski T., Weuve J., Bellinger D.C., Schwartz J., Lanphear B., Wright R.O. (2012). Cadmium exposure and neurodevelopmental outcomes in U.S. children. Environ. Health Perspect..

[9] Chakravartty D., Wiseman C.L.S., Cole D.C. (2014). Differential environmental exposure among non-Indigenous Canadians as a function of sex/gender and race/ethnicity variables: A scoping review. Can. J. Public Health.

[10] Martin D., Glass T.A., Bandee-Roche K., Todd A.C., Shi W., Schwartz B.S. (2006). Association of blood lead and tibia lead with blood pressure and hypertension in a community sample of older adults. Am. J. Epidemiol..

[11] Nash D., Magder L., Lustberg M., Sherwin R.W., Rubin R.J., Kaufmann R.B., Silbergeld E.K. (2003). Blood lead, blood pressure, and hypertension in perimenopausal and postmenopausal women. JAMA.

[12] Olsson I.-M., Bensryd I., Lundh T., Ottosson H., Skerfving S., Oskarsson A. (2002). Cadmium in blood and urine—Impact of sex, age, dietary intake, iron status, and former smoking—Association of renal effects. Environ. Health Perspect..

[13] Meltzer H.M., Alexander J., Brantsæter A.L., Borch-Iohnsen B., Ellingsen D.G., Thomassen Y., Holmen J., Ydersbond T.A. (2016). The impact of iron status and smoking on blood divalent metal concentrations in Norwegian women in the HUNT2 Study. J. Trace Elem. Med. Biol..

[14] World Health Organization (WHO) (2015). The Global Prevalence of Anaemia in 2011.

[15] Buettner C., Mukamal K., Gardiner P., Davis R., Phillips R., Mittleman M. (2009). Herbal supplement use and blood lead levels of United States adults. J. Gen. Intern. Med..

[16] Al-Saleh I., Al-Enazi S., Shinwari N. (2009). Assessment of lead in cosmetic products. Reg. Toxicol. Pharm..

[17] Cristaudo A., D’Ilio S., Gallinella B., Mosca A., Majorani C., Violante N., Senofonte O., Morrone A., Petrucci F. (2013). Use of potentially harmful skin-lightening products among immigrant women in Rome, Italy: A pilot study. Dermatology.

[18] Caravanos J., Gutierrez L.H., Ericson B., Fuller R. (2014). A comparison of burden of disease from toxic waste sites with other recognized public health threats in India, Indonesia and the Philippines. J. Health Pollut..

[19] Chatham-Stephens K., Caravanos J., Ericson B., Landrigan P., Fuller R. (2014). The pediatric burden of disease from lead exposure at toxic waste sites in low and middle income countries. Environ. Res..

[20] Janjua N.Z., Delzell E., Larson R.R., Meleth S., Kabagambe E.K., Kristensen S., Sathiakumar N. (2008). Maternal nutritional status during pregnancy and surma use determine cord lead levels in Karachi, Pakistan. Environ. Res..

[21] Lee Y.A., Hwang J., Kim H., Kim K.N., Ha E., Park H., Ha M., Kim Y., Hong Y., Chang N. (2013). Relationship between maternal sodium intake and blood lead concentration during pregnancy. Br. J. Nutr..

[22] Hsu C., Liu P., Chien C., Chou S., Han B. (2006). Mercury concentration and fish consumption in Taiwanese pregnant women. BJOG.

[23] You C.H., Kim B.G., Kim Y.M., Lee S.A., Kim R.B., Seo J.W., Hong Y.S. (2014). Relationship between dietary mercury intake and blood mercury level in Korea. J. Korean Med. Sci..

[24] Park S., Lee B. (2013). Strong positive associations between seafood, vegetables, and alcohol with blood mercury and urinary arsenic levels in the Korean adult population. Arch. Environ. Contam. Toxicol..

[25] Ikeh-Tawari E., Anetor J., Charles-Davies M. (2013). Cadmium level in pregnancy, influence on neonatal birth weight and possible amelioration by some essential trace elements. Toxicol. Int..

[26] Yuan X., Wang J., Shang Y., Sun B. (2014). Health risk assessment of cadmium via dietary intake by adults in China. J. Sci. Food Agric..

[27] Alba A., Carleton L., Dinkel L., Ruppe R. (2012). Increased lead levels in pregnancy among immigrant women. J. Midwifery Women Health.

[28] Miranda M.L., Edwards S., Maxson P.J. (2011). Mercury levels in an urban pregnant population in Durham County, North Carolina. Int. J. Environ. Res. Public Health.

[29] Scinicariello F., Abadin H.G., Murray H.E. (2011). Association of low-level blood lead and blood pressure in NHANES 1999–2006. Environ. Res..

[30] Lee M.G., Chun O.K., Song O.W. (2005). Determinants of the blood lead level of US women of reproductive age. J. Am. Coll. Nutr..

[31] Hightower J.M., O’Hare A., Hernandez G.T. (2006). Blood mercury reporting in NHANES: Identifying Asian, Pacific Islander, Native American, and multiracial groups. Environ. Health Perspect..

[32] Jain R.B. (2013). Effect of pregnancy on the levels of blood cadmium, lead and mercury for females aged 17–39 years old: Data from National Health and Nutrition Examination Survey 2003–2010. J. Toxicol. Environ. Health A.

[33] Mahaffey K.R., Clickner R.P., Bodurow C.C. (2004). Blood organic mercury and dietary mercury intake: National Health and Examination Survey, 1999 and 2000. Environ. Health Perspect..

[34] Mahaffey K.R., Clickner R.P., Jeffries R.A. (2009). Adult women’s blood mercury concentrations vary regionally in the United States: Association with patterns of fish consumption (NHANES 1999–2004). Environ. Health Perspect..

[35] Razzaghi H., Tinker S.C., Crider K. (2014). Blood mercury concentrations in pregnant and nonpregnant women in the United States: National Health and Nutrition Examination Survey 1999–2006. Am. J. Obstet. Gynecol..

[36] Mijal R.S., Holzman C.B. (2010). Blood cadmium levels in women of childbearing age vary by race/ethnicity. Environ. Res..

[37] Curren M.S., Davis K., Liang C.L., Adlard B., Foster W.G., Donaldson S.G., Kandola K., Brewster J., Potyrala M., Oostdam J.V. (2014). Comparing plasma concentrations of persistent organic pollutants and metals in primiparous women from northern and southern Canada. Sci. Total Environ..

[38] Foster W.G., Cheung A.P., Davis K., Graves G., Jarrell J., Leblanc A., Liang C.L., Leech T., Walker M., Weber J.P. (2012). Circulating metals and persistent organic pollutant concentrations in Canadian and non-Canadian born primiparous women from five Canadian centres: Results of a pilot biomonitoring study. Sci. Total Environ..

[39] Arksey H., O’Malley L. (2005). Scoping studies: Towards a methodological framework. Int. J. Soc. Res. Methodol..

[40] Andersen B., Blinder S. (2015). Who Counts as a Migrant? Definitions and Their Consequences.

[41] Thihalolipavan S., Candalla B., Ehrlich J. (2013). Examining pica in NYC pregnant women with elevated blood lead levels. Matern. Child Health J..

[42] Bakhireva L., Rowland A., Young B., Cano S., Phelan S.T., Artyushkova K., Rayburn W.F., Lewis J. (2013). Sources of potential lead exposure among pregnant women in New Mexico. Matern. Child Health J..

[43] Gulson B., Mizon K.J., Korsch M.J., Taylor A.J. (2006). Low blood lead levels do not appear to be further reduced by dietary supplements. Environ. Health Perspect..

[44] Rastogi S., Nandlike K., Fenster W. (2007). Elevated blood lead levels in pregnant women: Identification of high-risk population and interventions. J. Perinat. Med..

[45] Wu W., Liou S., Lin K., Liu T., Liu S., Chen C., Sung F., Wu T. (2009). Changing blood lead levels and DNA damage (commet assay) among immigrant women in Taiwan. Sci. Total Environ..

[46] Wu T., Wu C., Lin Y., Shen C., Liu T., Yang C., Liou S., Wu T. (2013). Changing blood lead levels and oxidative stress with duration of residence among Taiwan immigrants. J. Immigr. Minor. Health.

[47] Zhang W., Dewolf M., Hammadi S., Fris W., Noel E., Lorenzo R., Alexander S., The PLOMB 6 Group (2012). Lead levels in umbilical cord blood in Belgium: A cross-sectional study in five maternity units. Int. J. Hyg. Environ. Health.

[48] American College of Obstetricians and Gynecologists Committee on Obstetric Practice (2012). Lead Screening during Pregnancy and Lactation. Committee Opinions. http://www.acog.org/Resources-And-Publications/Committee-Opinions/Committee-on-Obstetric-Practice/Lead-Screening-During-Pregnancy-and-Lactation.

[49] Geer L.A., Persad M.D., Palmer C.D., Steuerwald A.J., Dalloul M., Abulafia O. (2012). Assessment of prenatal mercury exposure in a predominately Caribbean immigrant community in Brooklyn, NY. J. Environ. Monit..

[50] Soon R., Dye T.D., Ralston N.V., Berry M.J., Sauvage L.M. (2014). Seafood consumption and umbilical cord blood mercury concentrations in a multiethnic maternal and child health cohort. BMC Pregnancy Childbirth.

[51] United States Center for Disease Control and Prevention (U.S. CDC) (2009). Fourth National Report on Human Exposure to Environmental Chemicals. http://www.cdc.gov/exposurereport/pdf/fourthreport.pdf.

[52] Thomas M.R., Boekelheide K. (2013). Multiple environmental chemical exposures to lead, mercury and polychlorinated biphenyls among childbearing-aged women (NHANES 1999–2004): Body burden and risk factors. Environ. Res..

